# Annotations

**Published:** 1906-07-14

**Authors:** 


### ANNOTATIONS.

### The Buchanan Celebration in Scotland.

BORN in 1506 at Menteith, George Buchanan, the scholar, poet, wit, was the most famous Scotchman of his day, and for long afterwards. The period of his life—1506 to 1582—marks one of the most remarkable in the history of medicine. The first teacher of medicine in Scotland, Dr. James Cumyne, got ten marks a year from the Aberdeen University, which had then (1505) begun its medical faculty. In the same year (1505) anatomical practice was introduced into Scotland, and confirmed by a charter granted to the Royal College of Surgeons of Edinburgh by which they were enabled to dissect "ane hangit man" once a year. Andrew Vesalius was dissecting, and publishing his works, 1543. Ambrose Paré was taking the lead in the development of surgery in France, and in 1561 published the first complete system of surgery the world had ever seen. At this time George Buchanan was in France, drawn thither by "his old familiarity and the singular courtesy of that nation," teaching as Professor at Bordeaux University men like Montaigne and Scaliger. It was a great age—an age of culture and ascendency in which Scotland certainly in the domain of science and medicine was well in the front rank. Besides the stir in the Universities, Maister Peter Lowe, a pupil of Paré's, sharing his master's zeal for surgery and literature, founded at this time the Faculty of Physicians and Surgeons in Glasgow. The Scottish physician was the scholar and the philosopher in those days, and up to the end of the century in which George Buchanan lived, and for a long time afterwards, the Scottish intellect as a factor of European culture owes more to the Scot as a literary physician than to the Scot as a member of any other denomination. They occupied, like Buchanan, some of the highest professorial chairs abroad. Some of them, like Arthur Johnstone (1587-1641), even divided the honours for Latin literature with the famous Buchanan. Scotland had no Chaucer; no Shakespeare to mark the perfection of literary glory; no "Bernard of Gattesden and Gilbertin" to add lustre to medical literature—very few roses or other flowers, and not much even of happy thistle bloom. Yet there were marks of progress and culture and widening of intellect in Scotland, specially by the great names in medicine contemporaneous with George Buchanan and the era of the Scottish Reformation.

### Baths and Exercises in Cardiac Disease.

THE range and value of bath, gymnastic, and climatic treatment in cases of cardiac disease has provoked great differences of opinion, and more particularly in recent years. With the boom of the Nauheim "cult," some ten years or so ago, a great enthusiasm for active measures was developed, and this, without question, led to an indiscriminating excess that was in many cases harmful and even disastrous. Soon there followed the swing of the pendulum, and, as is usually the case in questions of therapeutic practice, it probably went too far in the opposite direction. Such is the penalty that attends exaggerated claims. The history of the Nauheim "cult" exactly illustrates this proposition, and its considerable decline during recent years is due largely to the excess of zeal of its early and injudicious advocates. The time now seems to have arrived when something like a confident position can be defined, and a very successful attempt to achieve this may be found in the address delivered during last session to the Balneological Society by Dr. Alexander Morison. In this it is urged that the gymnastic and bath treatment, though of real value in suitable cases, has but a limited field of application. This, briefly, includes (1) patients below middle age in whom there is moderate weakness of the myocardium, with or without irregularity, and either without valvular lesion or with a lesion other than aortic reflux, disease of the kidneys and pericardial adhesions being contra-indications; (2) older patients with myocardial debility but without valvular disease, and free from organic disease in other organs, from the symptoms of true angina, and from pronounced arterio-sclerosis. Altogether Dr. Morison's address is a valuable presentation of a difficult question, and it may well be consulted by those who are called upon to accept responsibility for the treatment of cases of chronic cardiac disease.

### The New Spa at Cheltenham.

THE enterprise of the Corporation of Cheltenham in establishing a Central Spa with a view to the encouragement of a more extended use of the various mineral waters for which the town has long been celebrated, will command the sympathy of all interested in the prosperity of our British health-resorts. Something, it is true, must doubtless be allowed for the decrees of custom and fashion, but these are not the only influences which cause the wide success of many Continental spas and the comparative neglect of home stations. In recent years there have been welcome evidences that, at least in some degree, those in authority at various British resorts have begun to realise that the mere possession of an abundant supply of a natural water having a more or less definite medicinal value is not alone sufficient to attract those in search of health. To this possession must be added some recognition of the necessity for business method and organisation, and for the provision of facilities for such reasonable exercise and entertainment as will promote gaiety of spirit and will avoid the depressing influences of stale, flat, and unprofitable monotony. The new spa at Cheltenham is emphatically a step in the right direction, and as such we gladly welcome it, and cordially wish it a large success. It is noteworthy that the waters of the district are not restricted to one type, but that, in addition to those of alkaline composition, there are others in which magnesium sulphate and sodium sulphate take a prominent place. When to these natural advantages there are added all modern hydro-therapeutic facilities and the charm of beautiful scenery and surroundings, Cheltenham ought to be recognised as a health-resort which has a wide claim for recognition, and one which need not fear comparison even with the most popular of its rivals.

